# Stability of the Nine Sky Quality Meters in the Dutch Night Sky Brightness Monitoring Network

**DOI:** 10.3390/s150409466

**Published:** 2015-04-22

**Authors:** Peter den Outer, Dorien Lolkema, Marty Haaima, Rene van der Hoff, Henk Spoelstra, Wim Schmidt

**Affiliations:** 1 National Institute for Public Health and the Environment, A. van Leeuwenhoeklaan 9, 3720 BA Bilthoven, The Netherlands; E-Mails: dorien.lolkema@rivm.nl (D.L.); marty.haaima@rivm.nl (M.H.); rene.van.der.hoff@rivm.nl (R.H.); 2 Lumineux Consult, Landgraafstraat 96, 6845 ED Arnhem, The Netherlands; E-Mail: henk@lumineux-consult.com; 3 Sotto le Stelle, Merwedeplantsoen 27, 3522 JS Utrecht, The Netherlands; E-Mail: wim.schmidt@sotto.nl

**Keywords:** artificial lighting, inter-comparison, inter-calibration, light pollution, night sky brightness, Sky Quality Meter

## Abstract

In the context of monitoring abundance of artificial light at night, the year-to-year stability of Sky Quality Meters (SQMs) is investigated by analysing intercalibrations derived from two measurement campaigns that were held in 2011 and 2012. An intercalibration comprises a light sensitivity factor and an offset for each SQM. The campaigns were concerned with monitoring measurements, each lasting one month. Nine SQMs, together forming the Night Sky Brightness Monitoring network (MHN) in The Netherlands, were involved in both campaigns. The stability of the intercalibration of these instruments leads to a year-to-year uncertainty (standard deviation) of 5% in the measured median luminance occurring at the MHN monitoring locations. For the 10-percentiles and 90-percentiles, we find 8% and 4%, respectively. This means that, for urban and industrial areas, changes in the sky brightness larger than 5% become detectable. Rural and nature areas require an 8%–9% change of the median luminance to be detectable. The light sensitivety agrees within 8% for the whole group of SQMs.

## 1. Introduction

The Night Sky Brightness Monitoring network in The Netherlands (MHN) consists of nine Sky Quality Meters (SQM-LE) that measure zenith night sky brightness. Measurements are used to gain insight into the differences in night sky brightness for different locations, e.g., nature reserve areas, rural and urban locations, and green house industry in The Netherlands, and to understand the observed differences in night sky brightness for different meteorological conditions. A next goal will be to determine if night sky brightness changes over time.

Artificial lighting may form threads to the natural dark nights and have impact on ecological and human health [1,2,3,4]. In order to preserve the quality of the natural dark night and minimise possible negative effects, knowledge is necessary on the geographical spread of night sky brightness levels and the development over night from evening to early morning. Carrying out measurements over longer periods of time, *i.e.*, years, offers the opportunity to monitor trends in night sky brightness and identify changes in the surrounding light emission.

The brightness level is highly depending on the local lighting, where “local” stretches to tens of kilometres. At any particular location, the bandwidth of the occurring luminance is mainly due to cloudiness. At areas with much artificial lighting, the night sky brightness is higher during overcast than under a clear sky, as clouds reflect the emitted upwards light downwards again. The lowest brightness levels are measured at cloud free nights [5,6]. This behaviour is reversed at locations that are remote and have little lighting. There, clouds shield the luminance due to the starry sky.

Although SQM devices were firstly developed to assess the night sky brightness for astronomical considerations, they have gained a second use as monitoring instruments to establish (abundance of) nighttime luminance and regional variability. Low cost and the relative ease with which these devices can be implemented in a monitoring network promoted this second use. A number of night sky brightness networks have been set up over the last few years, many of them exploiting Sky Quality Meter (SQM) devices [6,7,8,9,10,11,12]. Calibration of each SQM is performed by the manufacturer Unihedron [13] (Grimsby, ON, Canada). Giacomelli and Giubbilini [14] developed a calibration method themselves. Both calibration methods use a single [14] or narrow-band [13] light source for the calibration.

To better compare different locations, the SQMs should be compared in their “natural environment”, meaning outside and with light levels that occur at the different locations. Repeating such an inter-comparison over time offers the opportunity to identify the stability of the SQM devices under monitoring conditions and to compensate for instrumental drift.

Prior to installation of the SQM in the MHN, all nine were inter-compared during the Kick-Off Inter-comparison SQMs Campaign (KOIS) in 2011. This was repeated after a year with the International Cabauw Lightmeter Inter Comparison (CLIC) Campaign in 2013. Within the Loss of the Night Network (LoNNe) consortium, yearly SQM inter-comparison campaigns are scheduled [15]. In 2013, an inter-comparison campaign has been conducted in Lastovo, Croatia in 2013 [16]. In July 2014, a three-night inter-comparison campaign was conducted in Madrid, Spain [17]. 

In this paper, we analysed the year-to-year stability of SQMs in a monitoring environment by comparing the results of the nine Dutch SQMs involved in the two measurement campaigns KOIS and CLIC. 

## 2. KOIS 2011 and CLIC 2012

Both inter-comparison campaigns were held at the Cabauw Experimental Site for Atmospheric Research (CESAR) 51.9682°N, 4.9293°E in The Netherlands [18]. KOIS took place in 2011 from 1 April to 8 May, and CLIC in 2012 from 10 April to 9 May. We adopted the astronomical night, solar elevation below −18°, to set the limits of the measurements. This means that the campaign dates are limited until 19 May at this location. Data measured at times when the moon was above the horizon are excluded. During both campaigns, the SQMs were set up at the premises within 5 m distance of each other and all pointed at zenith; a residual inaccuracy of about 5° can, however, not be eliminated due to the housing of the devices. The aperture of an SQM is approximately 20° (full width at half maximum) as was established in our laboratory. The measurements during both campaigns were set to every 10 s. The 10 s data readings were “time-jitter” corrected and averaged to 1 min data readings before the rest of the analysis took place. The time-jitter has not an electronic origin, but is a consequence of small directional misalignments of the SQMs. Isolated clouds on an overall clear night give rise to an increased luminance when they drift over the site. An SQM facing upwind will detect the reflected light at a slightly earlier time than an SQM facing downwind. The overall effect results in readings from different SQM that appear to be shifted in time.

In the rest of the paper, all the 1-min readings produced by one particular SQM, limited by sun and moon elevation angles, and campaign dates will be termed “the data set” of that SQM for that campaign. It covers in total 35 nights for KOIS and 23 nights for the official CLIC campaign. One instrument owned by RIVM arrived too late to be included in the official campaign period. Therefore, the measurement period of CLIC was extended and an additional nine nights were captured (mostly by the Dutch SQMs).

An SQM uses a light-to-frequency IC (TSL237) that outputs a frequency directly proportional to the light intensity, combined with a Universal Frequency-to-Digital Converter. See [19] for more technical background. The wavelength responsivity is close to spectral response of the human eye. This output is digitally converted to magnitude per square arcsecond. The relationship between measured light intensity expressed as a Luminance *L* of the sky and magnitude per square arcsecond *m* is given by [13]:

*L [cd/m^2^]* = 10.8 × 10^4^ × 10^−0.4*m*^ (1)

The CLIC campaign also included measurements from eight IYA lightmeters and one DigiLum. The IYA lightmeters were installed by the University of Vienna [20]. These instruments have a wide field of view and a spectral sensitivity that is different to those of the SQMs. The DigiLum is a calibrated, highly sensitive photopic measuring device and has in contrast a narrower field of view. The DigiLum joined both inter-comparison campaigns.

**Figure 1:**
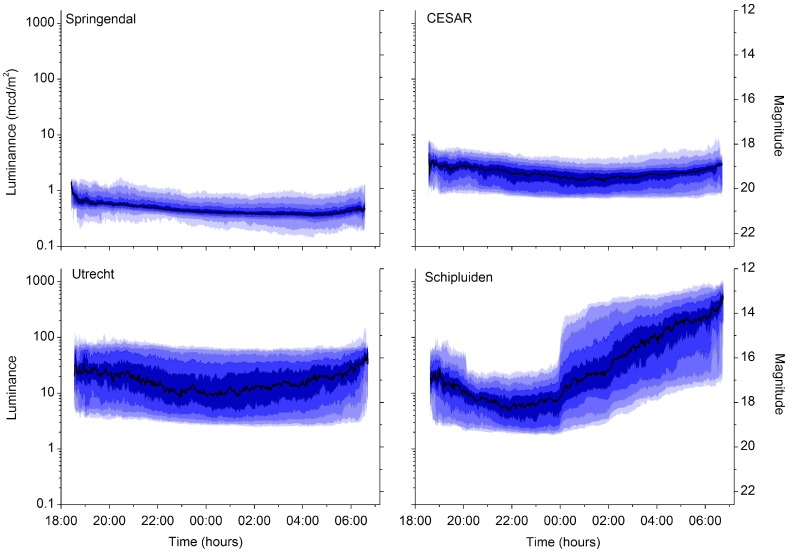
Luminance as function of time at four locations of the MHN. Moonlit nights are excluded, solar elevation is below −18°. The right axis gives the corresponding magnitude per square arcsecond. The median is indicated by the black line, and the darkest band is limited by the 60 and 40 percentile values. Bands have brighter colours towards smaller percentiles (30, 20, 10 and 5), or towards greater percentiles (70, 80, 90 and 95). Shielding of greenhouses is mandatory until midnight (Schipluiden).

The CESAR experimental site is owned and maintained by the Royal Netherlands Meteorological Institute, KNMI, and home to the CESAR-consortium, a consortium of eight partners that perform advanced atmospheric research. A large number of atmospheric measurements are performed at the same location. CESAR is located near Cabauw in the western part of the Netherlands. The surrounding landscape is open and consists of pasture and small villages. Larger cities are located at a distance of 20 km, Utrecht and 30 km, Rotterdam. The closest illuminated motorway (A27) is located at a distance of 6 km. The luminance at CESAR is illustrated in Figure 1.

We have plotted the percentiles as a function of the time during the night. The median has the darkest colour; each 10-percentile band away from the median has a lighter tint. We have added the measurements of three MHN-sites to illustrate the dynamical range observed in the MHN: a nature reserve area (Springendal), an urban location (Utrecht) and a location surrounded by greenhouse industry (Schipluiden). The pattern in the measured luminance at Schipluiden is a direct consequence of legislation; only the first half of the night is protected and depending on the initial light emissions, up to 95% of the emissions should be shielded. After midnight, covers are removed and windows opened to have a better indoor air and temperature regulation.

Figure 1 shows that the night sky brightness differs substantially within the MHN. As already state in the introduction: a measurement campaign intended to better assess the differences between the monitoring locations should covered the ranges of occurring luminance in the network as much as possible. At CESAR, the luminances are in the middle and not at the extreme (either very dark hence low luminances or bright meaning high luminances). This was an additional reason to host the campaigns at CESAR.

## 3. Inter-Comparison Results

One of the goals of the inter-comparison was to infer inter-calibration factors for the SQMs. Analysing the results of the KOIS and CLIC gives insight in the stability of the devices, in particular the nine Dutch SQMs that were included in both campaigns. The key to the used SQM labels is given in Table 1. It holds for both campaigns.

**Table 1:** Key to SQM numbers.

Label	Instrument ID	Owner	Default Location
SQM1	SQM987	RIVM	Arkemheen, The Netherlands
SQM2	SQM1707	RIVM	Schipluiden, The Netherlands
SQM3	SQM980	RIVM	Springendal, The Netherlands
SQM4	SQM1701	RIVM	Vlaardingen, The Netherlands
SQM5	SQM1368	RIVM	RadioKootwijk, The Netherlands
SQM6	SQM1366	RIVM	Schiermonnikoog, The Netherlands
SQM7	729_SLS	Sotto le stelle	Utrecht, The Netherlands
SQM8	SQM2_LC	Lumineux-consult	Arnhem, The Netherlands
SQM9	SQM1_LC	Lumineux-consult	CESAR, The Netherlands
SQM10	SQM1854	RIVM	Bilthoven/spare, The Netherlands
SQM11	SQM1760	Institut für Umweltphysik	Bremen, Germany
SQM12	SQM1710	Buiometria Partecipativa	Castiglioncello, Italy
SQM13	SQM849	Institute for Space Sciences	Berlin, Germany
SQM14	SQM828	Institute for Space Sciences	Berlin Germany
SQM15	SQM1865	Gruppo Astrofili Deep Sky	Brescia, Italy

Generally, during an inter-comparison the measurements of the instruments are compared to a reference. An external reference instrument can be used if applicable, or one instrument can be taken as the standard, or, a reference can be constructed using the measurements of all instruments included in the inter-comparison. During KOIS and CLIC the latter was done. An algorithm was adopted from Ultraviolet Radiospectrometer inter-comparisons to construct the reference. It puts higher statistical weight to mutual stable instruments, while the contribution of unstable instruments and outliers are suppressed. More details can be found in Slaper [21], and its application to SQM measurement during the KOIS campaign can be found in Den Outer [22]. The constructed reference inherits the same time resolution as the original data sets, *i.e.*, one value every minute in this case.

The sought inter-calibration factors for the offset (dark “current”) *C*_0_, and the slope *C*_1_ (light sensitivity or calibration factor) are delivered by a first order polynomial to a scatter plot of the reference as function of the data set of each SQM. The corrected measurements *L_cor_* are then given by:

*L_cor_* = *C*_0_ + *C*_1_*L* (2)

with *L* being the measured uncorrected Luminance. The obtained inter-calibration factors are listed in Table 2.

The inter-calibration factors found for the CLIC campaign show a much larger variability than found during the KOIS campaign. Moreover, the values for *C*_0_ are such that it would imply negative luminance values in the monitoring environment. Applying these numbers for the Dutch SQMs would suggest a 50%–100% calibration drift in one year. Both are rather unexpected. Although we do not have reasons to doubt the reference-generating algorithm, it is highly unlikely that the obtained numbers represent a true determination of the inter-calibration factors and long-term stability.

**Table 2:** Inter-calibration coefficients for offset *C*_0_ and slope *C*_1_ as first derived.

	C_0_ (mcd/m^2^)	C_1_
SQM1	0.2802 ± 0.002	1.0169 ± 0.0011
SQM2	0.0128 ± 0.003	0.9738 ± 0.0013
SQM3	0.2787 ± 0.001	0.9656 ± 0.0007
SQM4	−0.0222 ± 0.002	0.9780 ± 0.0010
SQM5	−0.1414 ± 0.002	0.9136 ± 0.0008
* SQM6	-- ± --	-- ± --
SQM7	0.2516 ± 0.002	1.1735 ± 0.0010
SQM8	0.3580 ± 0.001	1.3603 ± 0.0010
SQM9	0.2944 ± 0.001	1.0835 ± 0.0008
SQM10	−0.1831 ± 0.002	0.9297 ± 0.0009
SQM11	−0.4456 ± 0.003	0.9632 ± 0.0011
SQM12	0.2894 ± 0.002	1.0430 ± 0.0009
SQM13	−0.1452 ± 0.003	0.9248 ± 0.0010
SQM14	−0.2175 ± 0.005	0.9299 ± 0.0016
SQM15	0.2837 ± 0.010	0.9970 ± 0.0026

* SQM6 joined just after the ending of CLIC 2012.

**Figure 2:**
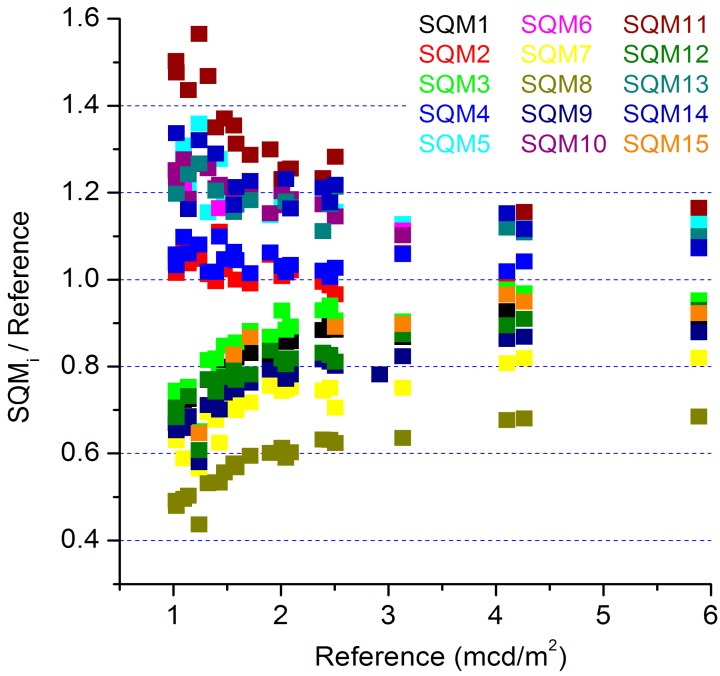
The average luminance per night for each SQM divided by the average derived from the reference is plotted as a function of the denominator.

**Figure 3:**
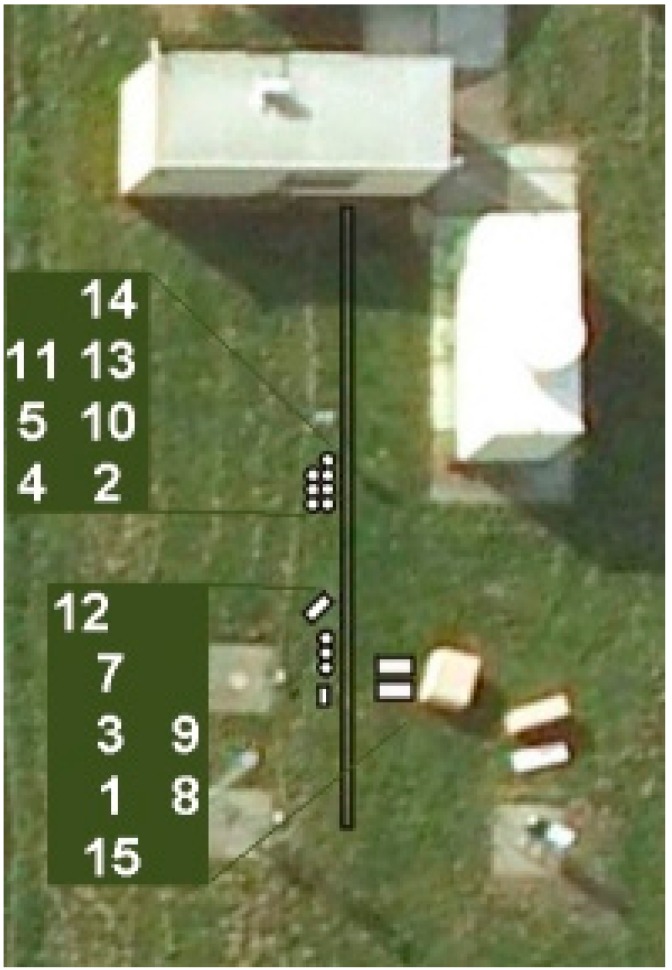
Top view of the CLIC instrument setup of the SQMs. Locations are indicated by white circles and rectangles. Insets show the corresponding numbers. As SQM 6 arrived late on the site and did not join the official CLIC-campaign period, it was placed at location 11 after the campaign.

Narrowing down the problem, we identified two groups that behaved differently at a low luminance. As is clearly shown in Figure 2, at the very dark nights the SQMs 2, 4, 5, 6, 10, 11, 13, and 14, hereafter referred to as group H (High), delivered higher values than the SQMs 1, 3, 7, 8, 9, 12, and 15, hereafter referred to as group L (Low). Figure 2 shows average luminance levels per night for each SQM divided by the same quantity according to the reference, plotted against this reference value. Most striking, the SQMs that belong to group H were placed relatively close to the Porto Cabin on the same rack, and the SQMs of group L were located further away, see Figure 3. Hence, these two groups were separated in place during the CLIC campaign.

**Figure 4:**
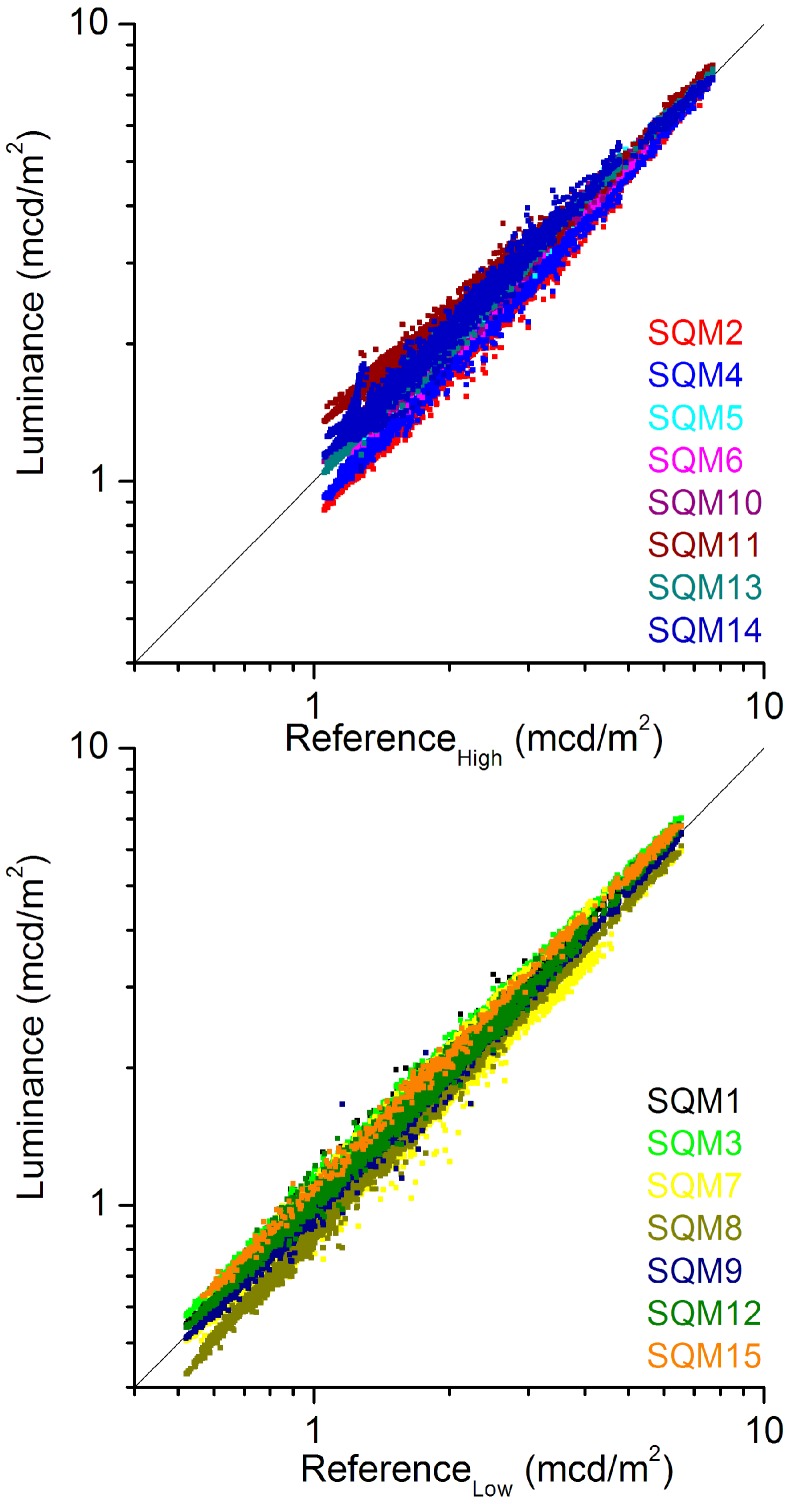
Top panel: measured luminance *vs.* reference for group H. Bottom panel: measured luminance *vs.* reference for group L.

Treating the two groups as separate, and thus calculating a reference for each group separately, does not only produce much better mutual agreement within the groups, but also points towards group H being the one who is affected. As Figure 4 shows at a low luminance, group H contains much larger differences between the individual SQMs and its reference, than found for group L. Therefore, we consider the readings by the SQMs of group H as being distorted, and those of group L to be valid. The offset in luminance, or the difference at low luminance, at the two locations is pinpointed by plotting the constructed reference for group H, *RH*, as a function of the reference for group L, *RL*, and performing a linear fit. This yields a rather large difference of 0.483 ± 0.001 mcd/m^2^.

Being aware of this artifact in the CLIC data sets, we re-analysed the KOIS campaign data in the same manner; the SQM were divided in two groups with respect to their location and the reference for each group separately was calculated. Group H was formed by SQMs 1 to 6, and group L contained 7, 8 and 9. This indeed revealed a similar behaviour but on a much smaller scale. For KOIS we determined a difference of only 0.035 ± 0.001 (mcd/m^2^) between *RH* and *RL*, hence over a factor 10 smaller than during CLIC. Therefore, this artifact was overlooked in our first analysis as reported in Den Outer [22].

Unfortunately, we could afterwards not identify a cause for this artifact. Whether it was an actual residual illumination due to some nearby LED of any apparatus or an electronic interference, remains unclear. Visible in Figure 3 are blurred images of several other instruments and power distribution units located relatively close to either of the groups. However, none can be unambiguously pointed at as being the source of the interference. A true sky brightness difference for these two locations that are separated by only a few meters is highly unlikely. The raw data of show constant differences between the readings of the SQMs (both for the luminance and for magnitude per square arcsecond), *i.e.*, there is not a time dependency or high frequency signal therein. This means that we can rule out time-dependent or (reflection of) moving light sources. A single fixed light source is not likely to have caused the artifact either considering the angular response of the SQMs and that they are pointed at zenith, and additionally, the orientation and distance with respect to group H and L of possible supports to attach a light source that would induce the observed properties of the artifact. Moreover, visual inspection on the site afterwards did not reveal nearby LEDs, in fact none were visible. We conclude that most likely the SQMs were subject to an electronic interferences either mutually, or with an external source. 

## 4. Stability Analysis

The measurement campaigns KOIS and CLIC were set up with the intent to make a stability analysis of our monitoring instruments and to establish the level of agreement with other (international) operating instruments. These initial goals are now of course severely hampered by the encountered artifact. The artifact should be accounted for by subtracting a background value from the measurements of group H. We perform three slightly different procedures to find these correction values and analyse the full year-to-year stability for each chosen/possible procedure. Averaging these results should make the final stability analysis less dependent on the exact route followed and should yield an estimate for the remaining uncertainties in the end-results. The procedures are set up such that data sets of both campaigns are treated in the same manner.

We expect to obtain results that are more consistent when we use the measurements of all SQMs, thus not limiting the analysis ahead to the nine Dutch SQMs that were included in both campaigns. The three instruments included in group L for KOIS, SQMs 7, 8 and 9, forms too small a group. The whole concept of constructing a reference is built on the availability of a range of measurements and instruments. Fortunately, the offset was small during KOIS, thus we can allow for some extra relative uncertainty here. 

It is straightforward to subtract the found offset between *RH* and *RL* from the measurements of group H. The results using this correction are labelled **A**. The top panel of Figure 4 indicates however, that the data sets have different offsets, and thus required different correction values. Labelled **I** are the results where we made linear fits to scatter plots of group H data sets *vs.* *RL* to obtain a correction value for each data sets*.* Labelled **S** are the results where we use values that minimize the quadratic term of a second order polynomial fit to logarithmic values of a data set of group H plotted *vs.* the logarithmic values of the *RL* data set. The results without corrections are marked **N**. The determined offsets found by each correction procedure are given in Table 3, a significant offset was also determined for SQM8 using procedure **S**. These values should be subtracted from the data sets.

**Table 3:** Determined Offsets in (mcd/m^2^).

	KOIS			CLIC		
	A	I	S	A	I	S
SQM1	0.0346	0.0133	0.0237	0.0000	0.0000	0.0000
SQM2	0.0346	0.1745	−0.0163	0.4829	0.3231	0.3583
SQM3	0.0346	−0.0075	0.0174	0.0000	0.0000	0.0000
SQM4	0.0346	0.1101	−0.0718	0.4829	0.3465	0.3745
SQM5	0.0346	0.0203	0.0083	0.4829	0.5008	0.5063
SQM6	0.0346	0.0004	0.0295	0.4829	0.5510	0.4615
SQM7	0.0000	0.0000	0.0000	0.0000	0.0000	0.0000
SQM8	0.0000	0.0000	0.0000	0.0000	0.0000	−0.0170
SQM9	0.0000	0.0000	0.0000	0.0000	0.0000	0.0000
SQM10	-	-	-	0.4829	0.5308	0.4368
SQM11	-	-	-	0.4829	0.7780	0.8215
SQM12	-	-	-	0.0000	0.0000	0.0000
SQM13	-	-	-	0.4829	0.4872	0.4296
SQM14	-	-	-	0.4829	0.5726	0.5390
SQM15	-	-	-	0.0000	0.0000	0.0000

**Figure 5:**
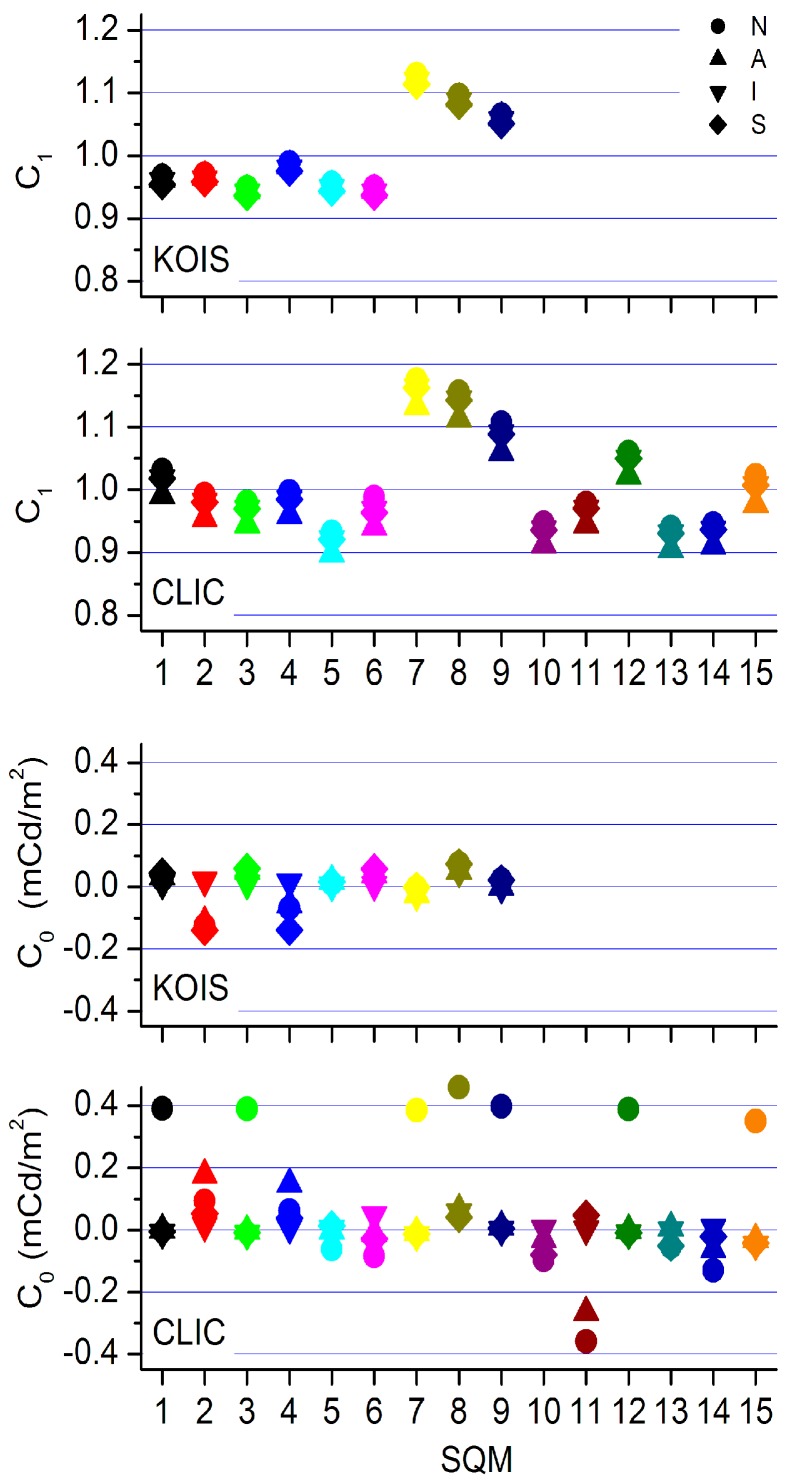
Slopes, *C*_1_ (**top panels**) and offsets *C*_0_ (**bottom panels**) are shown for all SQMs, corrections methods and both campaigns. Different symbols refer to different correction procedures, as indicated by the legend and explained in the text. **N**: no corrections, **A**: overall offset correction, **I**: offsets derived from linear fits, **S**: offsets yielding a minimum second order coefficient.

After implementing the offsets, the whole process of constructing a reference—using all data sets on an equal basis—and deriving the inter-calibration factors is carried out. This leads to a set of *C*_0_s and *C*_1_s for each SQM and for both campaigns, shown in Figure 5. We now find acceptable inter-calibration offsets and slopes. The extreme offsets, circle symbols that were mostly found in group L, are not present after group H is corrected. We also see that the offsets for SQM 7, 8, and 9 are almost the same for both campaigns. At the same time, we observe that the slopes are not particularly sensitive to the applied correction. The correction **A**, one offset correction for all group H members, produces the most aberrant slopes.

**Figure 6:**
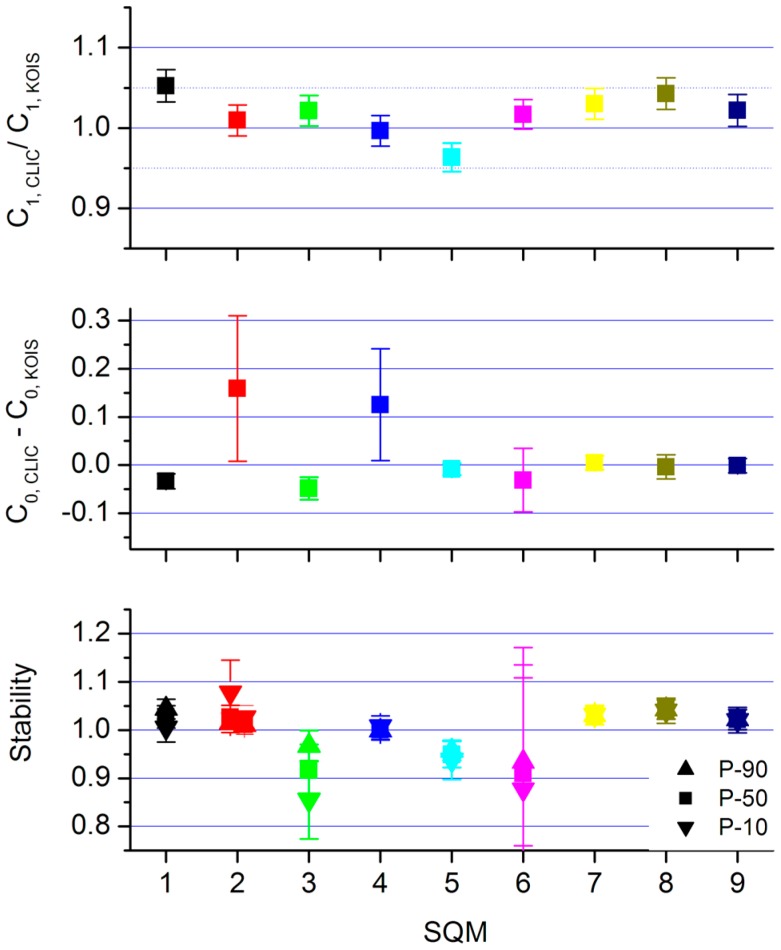
Year-to-year stability of the monitoring SQMs. **Top**: slopes. **Middle**: offsets, the unit is mcd/m^2^. **Bottom**: combined impact of slope-change and offset-drift on the 10-percentiles, median and 90-percentiles of the observed luminance at the monitoring locations.

In Figure 6, we show the derived year-to-year stability for the Dutch SQMs. We show averages of the outcome of the three correction procedures. The drift in offsets between CLIC and KOIS are averaged, and ratios *C*_1_s (KOIS) divided by *C*_1_s (CLIC) are averaged. The error bars indicate the standard deviation in the average, which we consider the best indication of the uncertainty in our analysis. The slopes are all within 5%—this means a magnitude per square arcsecond band of ∆m = 0.054 (≈0.05/0.4ln10)—the drift in the offsets are for most SQMs < 0.05 (mcd/m^2^), four SQMs have a negligible drift < 0.01 (mcd/m^2^). Only SQM 2 and 4 have larger drifts and uncertainties due to the underlying spread of the found offsets following the three correction procedures. These particular SQMs were already identified as the worst performing instruments in Den Outer [22].

The bottom panels shows the impact of the stability analysis on the median luminance levels as measured at each monitoring location. What is shown is the median level corrected for offset-drift and slope-change divided by the uncorrected value. The values for 10-percentiles and 90-percentiles are also shown. The combined impact of offset-drift and slope-change results in an uncertainty (standard deviation) of about 5% for the median, and 8% and 4% for the 10-percentiles and 90-percentiles, respectively. Due to the small luminance levels at the remote locations, the data of SQM3 and SQM6 are the most affected by their offset drifts. The measurements for Schipluiden (SQM2) are split in two: measured before and after midnight (the first is plotted at 1.9, the latter at 2.1).

Finally, we average the *C*_0_s and *C*_1_s of all three procedures for each SQM, and present the corrected version of the inter-calibration factors in Table 4. The indicated errors are the standard deviation in the averages. The *C*_0_s are small, less than a third of the starry sky background around 0.25 mcd/m^2^. The inter-calibration coefficients for KOIS are comparable to those derived in Den Outer [22], the main effect being an increase in the estimated error bars. This was to be expected because we deduced a small artifact for the KOIS campaign. The light sensitivity of the SQMs agree mutually within 7% (KOIS) and 8% (CLIC), *i.e.*, magnitude per square arcsecond bands ∆m ≈ 0.08 and ∆m ≈ 0.09 for KOIS and CLIC, respectively.

Our first effort had been to use the DigiLum instrument as the reference. However, the relationship between the DigiLum and SQMs proved to be luminance-dependent for small luminance. The luminance dependency is caused by differences in spectral response of SQM and DigiLum, and because of the apertures that differ for both instruments. Thus using the DigiLum as a reference would make the results rather sensitive to the distribution of luminance levels that have occurred.

**Table 4:** Inter-calibration coefficients for KOIS and CLIC, after correction.

	KOIS		CLIC	
	C_0_ (mcd/m^2^)	C_1_	C_0_ (mcd/m^2^)	C_1_
SQM1	0.03 ± 0.02	0.959 ± 0.004	−0.005 ± 0.005	1.009 ± 0.002
SQM2	−0.08 ± 0.09	0.963 ± 0.004	0.08 ± 0.09	0.972 ± 0.002
SQM3	0.04 ± 0.03	0.941 ± 0.004	−0.008 ± 0.005	0.962 ± 0.001
SQM4	−0.06 ± 0.08	0.980 ± 0.004	0.07 ± 0.07	0.976 ± 0.002
SQM5	0.01 ± 0.01	0.948 ± 0.004	0.005 ± 0.008	0.914 ± 0.001
SQM6	0.03 ± 0.03	0.942 ± 0.004	0.00 ± 0.04	0.958 ± 0.003
SQM7	−0.02 ± 0.03	1.119 ± 0.005	−0.013 ± 0.005	1.153 ± 0.003
SQM8	0.06 ± 0.01	1.087 ± 0.005	0.05 ± 0.01	1.133 ± 0.001
SQM9	0.01 ± 0.01	1.056 ± 0.005	0.005 ± 0.005	1.079 ± 0.001
SQM10	-	-	−0.035 ± 0.003	0.928 ± 0.001
SQM11	-	-	−0.07 ± 0.04	0.962 ± 0.002
SQM12	-	-	−0.1 ± 0.2	1.041 ± 0.002
SQM13	-	-	−0.015 ± 0.032	0.922 ± 0.002
SQM14	-	-	−0.03 ± 0.04	0.928 ± 0.003
SQM15	-	-	−0.04 ± 0.01	0.997 ± 0.003

## 5. Recommendations

We strongly recommend the repetition of inter-comparison campaigns with SQMs. So far, three inter-comparison campaigns with SQMs have been conducted. To access the quality of an operational network, the dynamical range of the luminance at the inter-comparison location should match the occurring levels at the network locations. Preferably, such an inter-comparison should be repeated yearly. In this way, a good understanding of the stability of the SQM devices over time can be established, which is a prerequisite for monitoring the night sky brightness. Good practice would be to include dark current measurements, *i.e.*, SQMs light tight covered, to detect and overcome the artifact we encountered. Hopefully, the LoNNe consortium will be able to do this with still two more inter-comparison campaigns (2015 and 2016) coming up. In addition, a good SQM calibration device, travelling from site to site, could help to a better overall comparability off monitoring locations. Alternatively, a well-calibrated travelling instrument with a high stability could be used that measures collocated for a short period, 1 to 2 weeks, at each monitoring locations.

## 6. Conclusions

We analysed the year-to-year stability of SQMs in a monitoring environment by comparing two measurement campaigns. Only SQM-measurements were used. The year-to-year stability of the light sensitivity has a standard deviation 5%, a magnitude per square arcsecond band of ∆m = 0.054, the drift in the offset is typically <0.05 (mcd/m^2^). Both can be considered a good result. It means that changes in the sky brightness larger than 5% become detectable for urban and industrial areas. At remote locations, with little light pollution, the detection limit is higher due to uncertainties in the offsets. The established drifts in the offset are probably an upper limit, and it is likely that they are in fact smaller. This part of the analysis was severely hampered by an artificial offset in the readings that occurred in half of the group of instruments. The whole group of SQMs have a light sensitivity that agrees within 8%.
